# Bibliographical Notices

**Published:** 1847-12

**Authors:** 


### BIBLIOGRAPHICAL NOTICES.

*On the Causes and Treatment of Abortion and Sterility; being the result of an extended inquiry into the Physiological and Morbid conditions of the Uterus, &c., &c.* By JAMES WHITEHEAD, F. R. C. S. Surgeon to the Manchester and Salford Lying-in Hospital. London, 1847.

Diseases of the Uterus have, of late years, engaged the attention of British and American practitioners in no inconsiderable degree; and we think justly. In the economy of the female, so important a rôle does the uterus play, that every thing relating to its diseased conditions cannot but be regarded as of very great importance. Hence the number of treatises which, of late years, have appeared on this subject from the British press, not to speak of the translations from the French.

We well recollect the severe, if not abusive article, which appeared some nine or ten years since in a highly respectable British journal, against Balbirney, on the use of the speculum in uterine diseases. A great change, however, since that period is manifested in British practitioners and authors. Perhaps not a single book comes to us now from that country that does not speak of the speculum and its use as one of the legitimate means of the physician for forming his diagnosis. We are glad of all this. It shows an improved state of the medical mind; it affords proof that no means are to be discarded for accurately ascertaining the condition of any suffering organ; and we feel warranted in saying that, but for the speculum, no man could practice judiciously and properly in diseases of the vagina and uterus.

The above work contains 426 pages. Of these, 180 are upon the subject of menstruation; 159 upon the signs of pregnancy; 160 upon the causes of abortion, and the remaining 27 upon sterility.

Of the chapters upon menstruation and signs of pregnancy, it has not been our good fortune to see exactly the reason of publication; certainly not for occupying more than one-half of the volume. This part could have been omitted, we think, without detriment to the remaining portion of the work, though it would certainly have diminished the size of the volume. Still, these chapters are not without interest, and will bear reading.

In speaking of the function of the Ovaries, the researches of Lee, Bischoff and Raciborski, and others, are mentioned; but we find no mention of Pouchet of Rouen. This eminent physiologist, as long ago as 1835, taught the positive theory of the fecundation of the mammifera. His views, since published, are summed up in ten fundamental physiological laws and three accessory. Raciborski and Bischoff's subsequent labours are but confirmations of Pouchet's laws.

*Spurious Menstruation—Pseudo-Pregnancy.*

Three cases are given, together with the constitutional signs of this condition of the uterus. In all three, disease of the organ existed; and the author lays down the rule that it is invariably associated with a morbid state of parts situated external to the uterine cavity, generally of its cervix and labia, sometimes of a portion of the vaginal mucous membrane.

*Suppressed Menstruation.*

A few interesting cases under this head are given—at the close of one of them, in which severe hysteric symptoms were present, and which terminated fatally, the author remarks:

"Hysteric affections are generally said by writers to be unattended with danger. Speaking of this form of disease, a modern author (Watson) says: 'It is a dreadful announcement to have to make to a father or mother that their child is epileptic, whereas hysteria, though it is sufficiently distressing, is attended in nine hundred and ninety-nine cases out of a thousand, with no ultimate peril to body or mind.' On the contrary, my belief is, that a greater amount of irreparable mischief has its origin in this class of affections in the adult female, than in derangement of any other system of organs, not excepting those of digestion and assimilation."

We are not sure that this is not a little too broadly stated; nevertheless, we feel confident that, at the commencement of menstruation, and for some years afterwards, hysteric affections are not to be regarded as of no moment, especially if, while the catamenia become obstructed from any cause, hysteric symptoms manifest themselves, the patient is in danger; for menstrual metastasis to the brain and spinal marrow may ensue, a condition of things almost always fatal.

Chapter 4th.—*On the last Menstrual Crisis.*—Endo-uteritis the author considers as one of the diseases liable to affect women at this period; but it is also found at other periods of life, and is not unfrequently the cause of dysmenorrhœa.

By Endo-uteritis, we are to understand an inflammation of the lining membrane of the uterus in part, or entire.

"In cases of endo-uteritis the body of the uterus is somewhat enlarged and painful under pressure of the finger; the cervix is slightly hypertrophied, but not so painful as the upper part of the organ, and it is generally free from abrasion. The only evidence of the existence of the disease capable of being revealed by the speculum, is the presence of the bright red ring surrounding the verge of the os uteri, together with the escape therefrom of the characteristic fluid product, or of a small quantity of blood, which, by becoming incorporated with the vaginal mucous in its transit outwards, appears at the os externum in the form of sanies."

"The constitutional symptoms are, rigors and febrile excitation, which varies in intensity according to the severity of the local inflammation, or the peculiar constitutional susceptibility of the individual; lassitude; sense of fulness; weight and tenderness of hypogastrium; fixed pain in the inguinal region, occupying one or both sides; disordered digestion; irritable bladder; spinal irritation and convulsive affections."

*Treatment of this affection.* Leeches to the *hypogastrium*, or over the upper part of the sacrum; the exhibition of remedies to subdue the febrile action. When all febrile excitement is subdued, a more tonic plan should be pursued. Considerable benefit may be derived from injecting within the uterus, a weak solution of nitrate of silver in combination with ext. conii.

"R. Argent. Nitrat. gr. vj.

Ext. Conii ʒj.

Aq. destill. ℥j

Misce fiat injectio."

Or by the introduction of an ointment of the same material.

"R. Argent. Nitrat. gr. x.

Unguent Cetacei ℥ss.

Liquoris Plumbi ʒss.

Misce, fiat Unguent."

"The latter form is applied by means of a small piece of lint fixed upon the end of a long probe, and fastened to the handle of the iustrument with a thread, with a view of securing its safe withdrawal. Not only does no disturbance or discomfort of any kind ensue upon the employment of these local measures, but, on the contrary, a beneficial change is often evident after the first application; and I have witnessed the suspension of pain to be instantaneous. The operation of injection is done with the aid of the speculum, by means of a long syringe, similar to Clarke's female syringe, having a nearly straight tube, with but one hole at its extremity. If carefully managed, from one to two drams of the remedy (which may be composed of different materials, varied in form and strength according to circumstances) will pass within the organ at each operation, most of which returns almost immediately. The temperature of the fluid, previous to being used, should be raised to 90° F."

We confess we have read this mode of treatment with some misgivings. We have never as yet even ventured to inject fluid into the body of the uterus. It may be unnecessary caution on our part; still we have never done it, lest an inflammation should be set up which might baffle all remedial means.

We should certainly think the saving clause, "when all febrile excitement has become completely subdued," ought to be rigidly adhered to.

CHAPTER 5. *Signs of Pregnancy.* We pass over this chapter and come to

CHAPTERS 7, 8, 9. *On the Causes of Abortion.*—These are the most interesting chapters in the book.

Two general causes; 1, disease of the cervix uteri; and 2, a venereal taint, are by far the most influential causes in the production of abortion.

Out of 378 cases examined carefully, 275 arose from diseases of the lower part of the uterus. What are these diseases? The author enumerates them as follows: 1. Inflammation, and superficial erosion of the cervix. 2. Varicose ulcer. 3. Œdema of the cervix. 4. Fissurated ulcer of the cervix. 5. Endo-uteritis. 6. Follicular ulceration.

To our mind the importance of attending to diseases of the cervix, in cases of abortion, cannot be too highly valued. Any one who will run his eye over the works on midwifery and the journals of the day, will perceive how inadequate are the means proposed to overcome abortion in an individual liable to abort. Rest, opiate injections, separation a thoro, all aid in certain cases; but most assuredly in many they fail.

Convinced, as we now are, of the frequency of diseased os and cervix uteri in recurring abortion in the same individual, we always call in the aid of the speculum. We have known, in more than one instance, a pregnant woman to be afflicted with what was termed a flooding, for months; abortion has followed; and on examination afterwards we have found a highly irritable state of all the parts, even extending to the bladder; a red, ugly looking ulcer, extending into the os itself, and oozing out from it a bloody fluid.

In all the forms of ulceration, escharotics to the ulcerated surface are indispensably necessary, and, for the most part, an alterative course of mercury and sarsaparilla.

The author prefers leeching to the hypogastrium. We prefer leeches applied directly to the os uteri, and occasionally scarifications with a *sharp* scarificator, attached to a long handle.

The French practice of applying a hot iron to the part is not so painful as might be supposed, if done with tact and delicately; in some obstinate cases of ulceration it is a valuable agent, more terrible in appearance than in reality.

The author lays down the three following positions from the cases narrated in these chapters on abortion.

1. That what is commonly called ulceration of the cervix uteri may be the predisposing, as well as the immediately exciting cause of abortion.

2. That the purulent product of uterine ulceration, under some forms, at least, possesses virulent properties, capable of producing disease in another individual, or in another part of the same individual, by inoculation; and probably capable also, by being absorbed into the circulation of the same person, of materially disordering the fluids, and of creating thereby a peculiar susceptibility to disease.

3. That the application of caustics to the uterus, and the employment of other active measures which I have heard practitioners object to during pregnancy, as likely to endanger the well-being of the offspring, may not only be safely administered, but that they constitute in fact one of the principal means of securing both mother and child from danger.

Several cases are narrated of varicose ulcer of the cervix, and the appropriate treatment given. We have not room for more than the three points which the author lays down, and which, if proved by experience hereafter to be true, will clear up some heretofore anomalous cases of what is termed menstruation during pregnancy.

1. That menstruation during pregnancy is, for the most part, perhaps always, associated with an abnormal condition, generally with ulcerative disease of the uterus, requiring at all times active remedial treatment.

2. That hæmorrhage during pregnancy is not necessarily associated with an altered relation of the parts within the uterus, and, by timely care, need not interfere with the integrity of the ovum.

3. That menstruation, during the early periods of lactation, is not always natural menstruation, but that it is generally associated with morbid conditions which are amply adequate to the satisfactory explanation of the phenomenon; that secondary hæmorrhage is, in the majority of instances, not owing to imperfect contraction, or atomy of the uterine fibres; and that the discharge very probably proceeds, under these circumstances, not from the inner surface of the uterus, but from the diseased surfaces situated upon parts external to the cavity of the organ.

*Syphilis as a cause of Abortion.*

The author confesses that when he commenced his investigations on the subject of abortion, he had no idea that syphilis was so common a cause as he afterwards was induced to believe it to be.

He thinks, and, we doubt not, justly, that the notion entertained of late by some that syphilis can be radically cured without the aid of mercury, is the cause of many disastrous consequences. Individuals have supposed themselves rid of this fearful disease, when, in fact, it was only covered over, as it were; subsequently they have married, and consequences most unhappy have followed. All practitioners in large cities must have seen examples of this kind. Mercury is to syphilis, what quinine is to ague. Without its use, in genuine syphilis, we do not think an individual safe.

The local pathognomonic signs of the uterus in a venereal taint are,

1. Endo-cervicitis, or inflammation of the lining membrane of the cervix uteri.
2. A mottled or patchy appearance of the cervix.
3. Apthæ of the cervix.
4. Warts on the cervix or walls of the vagina.

The author, as one of the remedial means, highly extols the Rumex Hydrolapathum. "I have used it for several years extensively, both in public and private practice, with the most gratifying results, in cases where sarsaparilla had been given in large doses for many months together, without the slightest benefit."

Of Prolapsus Uteri it is said: "That prolapsus uteri can be effectually treated, and the position of the organ permanently restored, without the aid of the pessary, there exists not a doubt on my mind!" Would that this could be proved experimentally true! How much trouble, vexation, and disappointment at last, in this very affection, have not the fairest of our race suffered!

So far as our observation goes it confirms the following statement:

"The cases (prolapsus uteri) are generally accompanied with leucorrhœa, and with inflammation or ulceration of the cervix uteri, evidences of which are found in almost all instances to have prevailed at a period anterior to the occurrence of the displacement."

The treatment is to be directed locally to the diseased cervix, and generally to the constitution.

Of the first, nit. argent., or other suitable remedies; medicated tents are to be applied to the os, by means of a tube, and changed every one or two days. The metallic preparations should be used not oftener than every third or fourth day, vegetable applications, as matico, tannin, &c., being adopted intermediately.

Of the second, when all febrile excitement is absent, quinine, metallic oxides, iodine, iron combined with cinchona extract or taraxacum, gentian, &c.

Chapter 10. *Sterility.* It is true, as the author observes, that the cause of sterility is generally attributed to a faulty condition of the female organs. No doubt correctly. When we consider the complicated female genital apparatus, the chances are certainly vastly in favour of the faulty condition of this, as compared with that of the male.

The author, however, in this work proposes merely to treat of those cases of sterility, which result from a morbid state of the uterus, originally of a normal condition.

Three principal causes are mentioned, viz: 1. Endo-uteritis; 2. Spyhilis; and 3. Ulceration of the cervix.

The means employed in the treatment of the first and third, are such as have been already given—viz: local applications to the diseased surfaces, and constitutional treatment of an alterative and tonic character, as iodide of iron and sarsaparilla.

In some cases, according to Mr. Whitehead, the uterus has resumed its natural function, after an appropriate course of treatment; leading to the hope that as our knowledge advances we may be more and more enabled to overcome this abnormal state of the uterine function, the cause often of much unhappiness.

We trust that the views expressed by the author of this work, especially on the subject of abortion, will be more generally entertained and carried out in daily practice. We feel confident that if they do become more generally understood, and practised upon, much suffering and danger will be obviated, and relief and comparative health afforded to those who otherwise may be doomed to drag out a miserable existence. †

---

*The American Medical Almanac, for* 1848, *containing statistics of the various Medical Colleges, Hospitals, Dispensaries, etc., of the United States; together with other information of value to the Physician and Student.* 18mo. pp. 224. Lindsay & Blakiston, Philadelphia.

The title page of this publication indicates very truly the character of its contents. Beside the account of Colleges, Hospitals, Dispensaries, private associations for granting instruction on the various branches of Medical Science, the work contains the organization of the National Medical Association; the code of ethics adopted by the late National Medical Convention; a chapter on Poisons, their antidotes, and the treatment proper in cases in which the various poisons have been taken; several articles on the inhalation of ether; Dr. Burder's letters on the importance of promoting the religious welfare of patients, and various miscellaneous articles of general interest to medical men, making altogether an exceedingly interesting and useful volume.

